# Supplementary material for: Preparation and Swelling Behaviors of High-Strength Hemicellulose-g-Polydopamine Composite Hydrogels
Source: Materials (Basel). 2021 Jan 2;14(1):186. doi: 10.3390/ma14010186 (PMC7795248; doi:10.3390/ma14010186)
Supplement: Supplementary file 1 [file materials-14-00186-s001.pdf]

Supplementary Materials

Preparation and Swelling Behaviors of High-Strength Hemicellulose-g-Polydopamine Composite Hydrogels

Jiayan Ge [1], Kaiqi Gu [1], Kewen Sun [2], Xinyue Wang [1], Shuangquan Yao [1,*], Xiaorong Mo [1], Shuilian Long [1], Tingting Lan [1] and Chengrong Qin [1,*]

1 Guangxi Key Laboratory of Clean Pulp & Papermaking and Pollution Control, School of Light Industrial and Food Engineering, Guangxi University, Nanning, 530004, China; 1816391008@st.gxu.edu.cn (J.G.); 1805170140@st.gxu.edu.cn (K.Q.); 1805170104@st.gxu.edu.cn (X.W.); 858317445@qq.com (X.M.); longshuilian77@163.com (S.L.); lantingtingd@163.com (T.L.)

2 School of Computer & Information, Hefei University of Technology, Hefei, 230009, China; sunkewen2014@163.com

* Correspondence: yaoshuangquan@gxu.edu.cn (S.Y.); qinchengrong@gxu.edu.cn (C.Q.)

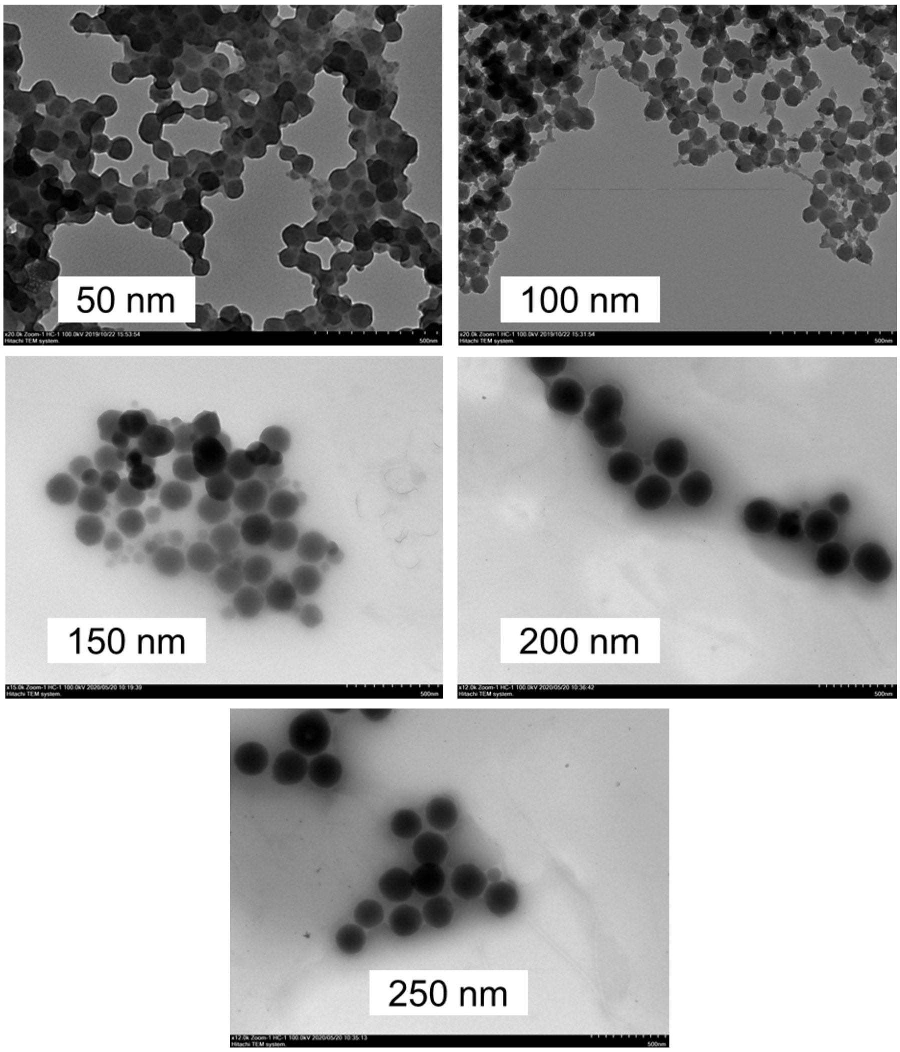

Figure S1. TEM of different polydopamine microsphere sample size.
